# Functional Assembly of *Caenorhabditis elegans* Cytochrome b-2 (Cecytb-2) into Phospholipid Bilayer Nanodisc with Enhanced Iron Reductase Activity

**DOI:** 10.3390/biom11010096

**Published:** 2021-01-13

**Authors:** Hamed A. Abosharaf, Yuki Sakamoto, Aliaa M. Radwan, Keisuke Yuzu, Mika Fujimura, Thoria Diab, Tarek M. Mohamed, Eri Chatani, Tetsunari Kimura, Motonari Tsubaki

**Affiliations:** 1 Department of Chemistry, Graduate School of Science, Kobe University, Nada-ku, Kobe, Hyogo 657-8501, Japan; ac_for_npi@yahoo.co.jp (Y.S.); alyaa_radwan@science.tanta.edu.eg (A.M.R.); 192s227s@stu.kobe-u.ac.jp (K.Y.); mika-fujimura@unicharm.com (M.F.); chatani@crystal.kobe-u.ac.jp (E.C.); tetsunari.kimura@people.kobe-u.ac.jp (T.K.); 2 Biochemistry Division, Chemistry Department, Faculty of Science, Tanta University, Tanta 31527, Egypt; thoria.diab@science.tanta.edu.eg (T.D.); tarek.ali@science.tanta.edu.eg (T.M.M.)

**Keywords:** *C. elegans*, Cecytb-2, ferric reductase, nanodisc, nitroso-PSAP

## Abstract

Among seven homologs of cytochrome *b*_561_ in a model organism *C. elegans*, Cecytb-2 was confirmed to be expressed in digestive organs and was considered as a homolog of human Dcytb functioning as a ferric reductase. Cecytb-2 protein was expressed in *Pichia pastoris* cells, purified, and reconstituted into a phospholipid bilayer nanodisc. The reconstituted Cecytb-2 in nanodisc environments was extremely stable and more reducible with ascorbate than in a detergent-micelle state. We confirmed the ferric reductase activity of Cecytb-2 by analyzing the oxidation of ferrous heme upon addition of ferric substrate under anaerobic conditions, where clear and saturable dependencies on the substrate concentrations following the Michaelis–Menten equation were observed. Further, we confirmed that the ferric substrate was converted to a ferrous state by using a nitroso-PSAP assay. Importantly, we observed that the ferric reductase activity of Cecytb-2 became enhanced in the phospholipid bilayer nanodisc.

## 1. Introduction

Cytochromes *b*_561_(CYB561) are a group of transmembrane proteins which distribute widely in many eukaryotic cells [1]. They have a unique structure including six hydrophobic transmembrane α-helices being bound with two heme *b* prosthetic groups [1,2]. The redox potential measurements for the purified proteins indicated that the cytosolic ascorbate is the electron donor for the CYB561 proteins [3,4]. Furthermore, some members of CYB561 showed a distinct ferric reductase activity that could have a vital role for the iron metabolism of eukaryotic cells [5]. Hence, further understandings of the CYB561 function and their roles in the iron metabolism can be achieved using animal models. A nematode *Caenorhabditis elegans (C. elegans)* has seven types of CYB561 homologs (Cecytb-1 -7). Among them, Cecytb-2 was confirmed to be expressed in its digestive organs and is homologous to human duodenal cytochrome *b* (Dcytb) (Miura et al., unpublished results).

Iron is a major metal in living organisms and exists in a ferric (Fe^3+^) state in the physiological aerobic conditions and is involved in various critical biological processes such as DNA synthesis, ATP production, and neurotransmitter synthesis [6,7]. Overload of iron in cells can produce free radicals through a Fenton reaction, causing severe cellular damages and neurodegenerative disorders [8]. Therefore, it is necessary to strictly control the iron concentration in the living body and maintain its homeostasis.

For the acquisition of iron into a body, a ferric ion is reduced to a ferrous state on the surface of duodenal cells by metalloreductase proteins and imported into the cytoplasm by divalent metal transporter-1 (DMT1) [6,9]. Previous studies revealed that a form of CYB561 has such a function. Indeed, human duodenal cytochrome *b*_561_ (Dcytb) was shown to have a ferric reductase activity for the transport of ferrous ions into intestinal duodenal mucosa cells [10]. Furthermore, it was reported that tonoplast cytochrome *b*_561_ in the *Arabidopsis thalina* sp. (TCytb) has an ascorbate-dependent ferric reductase activity [11], although its role in plant cells is not clarified yet. Other members of the mammalian cytochrome *b*_561_ family were also proposed to have a ferric reductase activity [9,12].

Detergent solubilization is a common method to study various membrane proteins in the solubilized state by utilizing the amphipathic nature of detergents. However, usage of mild detergents could nevertheless hamper their functions by altering their 3D structure or by dropping their stability [13]. Nanodisc (ND) is a newly introduced approach to study membrane proteins which are reconstituted into a phospholipid bilayer encircled by two molecules of a membrane scaffold protein (MSP) as a belt. NDs could overcome all undesirable effects of detergent by providing native-like environments for the membrane proteins [14,15] including cytochromes P450 and *b*_5_ [16,17,18,19,20]. Self-assembly of the membrane proteins in nanodiscs enables the usage of various biochemical and biophysical techniques for studying the structure and functions of the membrane proteins [21]. Reconstitution of nanodiscs in a controlled size depends on the length of the MSP and the stoichiometry among lipid, MSP, and the target protein, which should be determined by a series of experiments of nanodisc assemblies to find an optimal ratio [22,23].

In our previous study, we employed the nanodisc technology for the first time to reveal the enzymatic activity of cytochrome *b*_561_ protein family at the molecular level. We verified that purified human 101F6 protein has an authentic ferric reductase activity [23]. However, for other members of the cytochrome *b*_561_ protein family, the presence of the ferric resuctase activity was not proved yet on the molecular level. 101F6 protein differs significantly from Cecytb-2 protein (or other classic members of cytochrome *b*_561_ family) in amino acid sequences (belonging to a very distant subfamily) and in their biophysical properties (EPR spectra, redox potentials, reactivity with ascorbate) [24,25,26]. Therefore, the molecular mechanism of the ferric reductase activity of 101F6 may be very different from those of usual cytochromes *b*_561_.

In the current study, we succeeded in reconstituting purified Cecytb-2 protein into a phospholipid bilayer nanodisc and studied its spectral properties, thermal stability, and molecular size. Then, we studied on the ferric reductase activity of the Cecytb-2 protein in a nanodisc. We found that the ferric reductase activity of the Cecytb-2 as well as its protein stability was enhanced significantly in the nanodisc environments. These results suggested that the reconstitution into a nanodisc environment can provide very suitable environments for studying structure and functions of very hydrophobic proteins, like Cecytb-2.

## 2. Material and Methods

### 2.1. Materials

Plasmid (pET28a-MSP1D1ΔH5) used for the transformation of *E. coli* BL21(DE3) strain was purchased from Addgene (Watertown, MA, USA). Ferric ammonium citrate (FAC) was obtained from Wako Pure Chemical Industries, Ltd., Osaka, Japan. 2-Nitroso-5-[N-n-propyl-N-(3sulfopropyl) amino] phenol (Nitroso-PSAP) and Dodecyl-β-maltoside (DDM) were purchased from Dojindo Laboratories, Kumamoto, Japan. 1,2-Dimyristoyl-sn-glycero-3-phosphocholine (DMPC) was from Tokyo Chemical Industry, Tokyo, Japan. Bio-beads SM-2 was obtained from BIORAD, Hercules, CA, USA. Chromatographic columns were purchased from GE Healthcare Japan Ltd., Tokyo, Japan. All other chemicals were obtained in the highest grade.

### 2.2. Expression and Purification of Cecytb-2

The heterologous expression of Cecytb-2 protein was performed using methylotropic yeast *P. pastoris* cells (Miura et al., unpublished results). The linearized plasmid pPICZB-Cecytb-2-H6 with *Pme*I was inserted into the *P. pastoris* GS115 genome using EasyComp transformation protocol (Invitrogen Corp., Tokyo, Japan). Successfully transformed cells were selected on Yeast Extract Peptone Dextrose Sorbitol Medium (YPDS) agar plates containing 100–400 μg/mL Zeocin (Invitrogen Corp.) The culture for the Cecytb-2 expression was done as previously reported in [27], (Miura et al., unpublished results). The purification of the expressed Cecytb-2 was performed with Ni-Sepharose affinity column as described in [27,28] with some modification (for detailed procedures, see Supplementary file) (Miura et al., unpublished results). UV-visible absorption spectra of the purified Ceytb-2 protein were recorded in a region from 700 to 200 nm using a Shimadzu UV-2400PC spectrophotometer (Shimadzu Corp., Kyoto, Japan). The reducibility of Cecytb-2 was checked using 10 mM ascorbic acid (AsA) and sodium dithionite (with few grains, corresponding to about 50 mM). Total amounts of protein at every step of purification were determined using the Bradford method [29] with bovine serum albumin as a standard. Cecytb-2 protein were assessed as the cytochrome *b*_561_ content using an extinction coefficient of 39.47 mM^−1^cm^−1^ at 561 nm [30].

### 2.3. Reconstitution of Cecytb-2 into Nanodisc

MSP1D1ΔH5 (MW; 22.1 kDa) was hetrologously expressed using *E. coli* BL21(DE3) system and purified by Ni-NTA Sepharose affinity column chromatography [22,31]. Purified MSP1D1ΔH5 was mixed with DMPC/cholate mixture (50/100 mM in 20 mM Tris HCl, 100 mM NaCl, pH 7.4) to give the optimal ratio of 1:80. After 30 min agitation at room temperature, purified Cecytb-2 was added to give a molar ratio of 1: 80: 0.5. After 1 h of incubation at room temperature, detergent was eliminated by adding 120 mg of Biobeads SM-2 (BioRad, Hercules, CA, USA) for 16 h with gentle agitation at room temperature. Thus, prepared nanodiscs were filtered through a 0.22 µm filter and centrifuged at 15,000 rpm for 10 min at 4 °C to remove large aggregations. Then, the reconstituted nanodiscs were purified by Size Exclusion Chromatography (SEC) using Superdex^TM^ 200 10/300 GL column (AKTA pure chromatography system; GE healthcare Japan Ltd., Tokyo, Japan) [32]. The column was pre-equilibrated with elution buffer (20 mM Tris HCl, 100 mM NaCl, pH 7.4) and the nanodisc mixture was eluted using the elution buffer with a flow rate of 0.4 mL/ min by recording A_280_ for total protein and A_416_ for Cecytb-2, simultaneously [23]. The collected fractions in test tubes were further analyzed by UV-visible spectroscopy and by SDS-PAGE to evaluate the chemical properties and to quantify the physical composition of the purified Cecytb-2-nanodisc complex.

### 2.4. Homogeneity and Particle Size Distribution of Nanodisc

The homogeneity and apparent molecular size of the purified Cecytb-2-nanodisc were determined by dynamic light scattering (DLS) with a nanoparticle size analyzer (Zetasizer Nano analyzer; Malvern Analytical Ltd.; Malvern, UK) using a light source (632.8 nm) from a He-Ne laser. Firstly, the samples were incubated at room temperature for 1 h and then filtered through a 0.2 µm cellulose acetate filter. Then the samples were transferred into a PS cell and their DLS was measured at 633 nm with a scattering angle of 173° at 25 °C. The data were analyzed by Zetasizer Nano software (Malvern, UK) to give their number-based size distributions.

### 2.5. Thermal Stability of Cecytb-2 in a Nanodisc and in a Detergent Micelle State

Cecytb-2 proteins in DDM detergent micelle state (50 mM potassium phosphate buffer, 10% glycerol, 0.1% DDM, pH 7.4) or in nanodisc state (20 mM Tris HCl, 100 mM NaCl, pH 7.4) were incubated for 15 min at different temperatures (4 °C~75 °C). Then the samples were centrifuged briefly to remove turbidity and then reduced at room temperature by sodium dithionite followed by monitoring the UV-visible spectral change. The reduction level (%) was calculated using the following equation: % reduction level = (A_427_ − A_min_/A_max_ − A_min_) × 100; where A_min_ is the absorbance at 427 nm of the oxidized state of the native form and A_max_ is the absorbance at 427 nm of the reduced state (by sodium dithionite) of the native form. In the calculation, we assumed that there were only two chemical species (reduced and oxidized forms) in equilibrium in the reaction mixture. We further assumed that the precipitated parts by centrifugation could be regarded as a denatured non-reducible form.

### 2.6. Measurements of Ferric Reductase Activity of Cecytb-2

Removal of molecular dioxygen (or keeping it at a very low level) is a prerequisite process to evaluate the oxidation process of the reduced heme centers of Cecytb-2 (or its electron transfer to the ferric substrate; i.e., the ferric reductase activity). For evaluating the ferric reductase activity of Cecytb-2, we conducted it as follows (Fujimura et al., unpublished results). First, the auto-oxidation of reduced Cecytb-2 was measured in DDM-detergent micelle state and in nanodisc state by monitoring the decay of the absorbance peak at 427 nm of the ferrous *b*-type heme. Briefly, the measuring buffer (50 mM Tris HCl, 10% glycerol, pH 7.4) was bubbled with pure nitrogen gas for 1 h. Then, DDM was added to the buffer anaerobically to give a final concentration of 0.1%. Then, a HiTrap^TM^ desalting column was deoxygenated by pre-equilibrating with the deoxygenated measuring buffer. Then, Cecytb-2 reduced with sodium dithionite in an anaerobic tube was loaded onto the deoxygenated column and eluted anaerobically using the deoxygenated measuring buffer into a deoxygenated quartz cuvette. Then, UV-visible spectra were recorded continuously using a repeated scan mode of the spectrophotometer (UV-2400PC, Shimadzu Corporation, Kyoto, Japan) for 90 min. The autoxidation process of the reduced heme was fitted by a single exponential function; y = y_0_ + A_1_ × exp(−*k*_1_t). For the measurements of the ferric reductase activity, the spectral changes were recorded immediately after the anaerobic addition of ferric substrate (FAC) to the fully reduced form of Cecytb-2 using the repeated scan mode. Then the absorbance change at 427 nm was fitted using a double exponential function; y = y_0_ + A_1_ exp(−*k*_1_t) + A_2_ exp(−*k*_2_t). In the fitting process, one of the rate constants (*k*_1_) was fixed at the same value with that obtained for the heme autoxidation. In the case of the nanodisc state, the measurements were performed in the same manner but the measuring buffer without DDM was used throughout.

### 2.7. Measurements of Ferric Reductase Activity by Nitroso-PSAP Assay

Nitroso-PSAP chelates with Fe^2+^ and forms a Fe^2+^-nitroso-PSAP complex that displays a characteristic absorption peak at 756 nm with a molar absorptivity of 45 mM^−1^cm^−1^ [33]. By utilizing this nature, measurements of the ferric reductase activity of Cecytb-2 were conducted as follows (Fujimura et al., unpublished results). Briefly, spectral changes were continuously recorded in the repeated scan mode upon the anaerobic addition of 2 µM of FAC and 8 µM of nitroso-PSAP to the pre-prepared reduced form of Cecytb-2 (either in nanodisc state or in DDM-detergent micelle state) (2 µM) as previously described. The decay of the reduced heme at 561 nm and the formation of the Fe^2+^-nitroso-PSAP complex at 756 nm were plotted against time and these changes were fitted using a single exponential equation; y = y_0_ + A_1_ × exp(−*k*_1_t). A single exponential function was chosen just for the purpose of evaluating the apparent rates. All analyses and fitting were conducted using Igor Pro (v. 6.37).

## 3. Results

### 3.1. Cecytb-2 Purification and its Assembly into a Nanodisc

**Figure 1:**
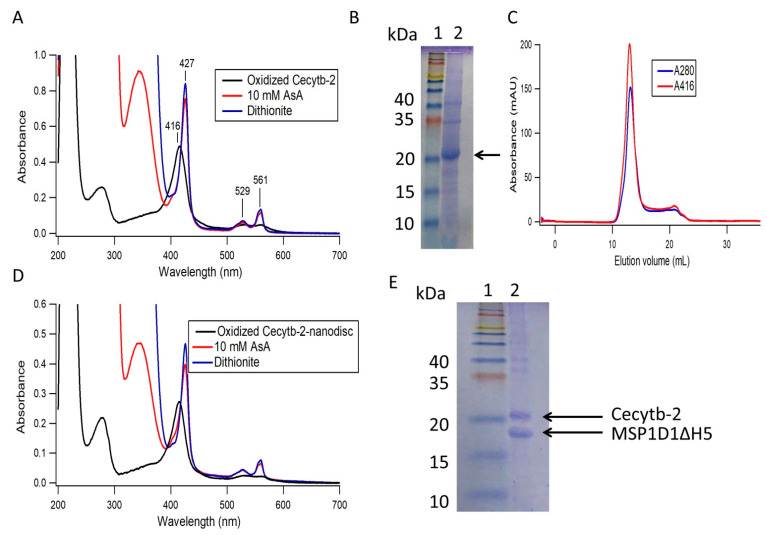
The purification of Cecytb-2 protein and its reconstitution into a nanodisc. (**A**) UV-visible absorption spectra of purified Cecytb-2 in an oxidized state (black line and in the reduced state by 10 mM AsA (red line) and ~50 mM sodium dithionite (blue line). (**B**) SDS-PAGE of Cecytb-2 after the Ni-affinity purification; lane (1) protein marker (Prestained XL-ladder, APRO Life Science Institute, Inc, Osaka, Japan); lane (2) Cecytb-2 eluted by the elution buffer containing 350 mM imidazole (black arrow). (**C**) The SEC of self-assembled Cecytb-2 into a phospholipid bilayer nanodisc; the red line shows the absorbance at 416 nm of the heme group of Cecytb-2 and the blue line shows the absorbance at 280 nm of total protein. (**D**) UV-visible spectra of the purified fractions of the Cecytb-2-nanodisc complex after the SEC purification; the black line shows the air oxidized state, red and blue lines indicate the reduced states with 10 mM ascorbic acid and with ~50 mM sodium dithionite, respectively. (**E**) SDS-PAGE of the purified Cecytb-2-nanodisc complex after SEC. Lane (1) protein marker (Prestained XL-ladder (SP-2140), APRO Life Science Institute, Inc, Osaka, Japan); lane (2) Cecytb-2 nanodisc (Cecytb-2, 29,2 kDa and MSP1D1ΔH5, 22.1 kDa).

In this study, we succeeded to express Cecytb-2 protein in *Pichia pastoris* cells as a fusion protein with a six-histidine–residue-tag at the C-terminus (Cecytb-2-His_6_). Then the Cecytb-2- His_6_ protein was purified using Ni-NTA Sepharose column by employing the high affinity of the histidine residue-tag towards the Ni^+2^ moieties. The purification steps indicated that the 1.5 L scale of the culture could produce 4.14 mg of Cecytb-2-His_6_ with a purification fold of 21.07 (Table 1). The UV-visible absorption spectrum of the oxidized Cecytb-2 protein indicated the presence of broad Q bands from 600 to 500 nm and a characteristic sharp Soret peak at 416 nm. Upon reduction with sodium dithionite, the broad Q band was sharpened and resolved into α and β bands at 561 nm and 529 nm respectively, and the Soret band shifted to 427 nm, characteristic properties of *b*-type heme moiety. Moreover, the spectral properties revealed that Cecytb-2-His_6_ protein was able to receive electrons from ascorbic acid (AsA) but the final reduction level was slightly lower than that with sodium dithionite (84.5% (± 7.8)) (Figure 1A), as observed for other cytochrome *b*_561_ family members [24,34]. The SDS-PAGE analysis showed a strong band close to the theoretical molecular mass of Cecytb-2-His_6_ (29,227 Da) (Figure 1B). It is noted that the Cecytb-2 band runs smaller than its expected molecular mass (~29.2 kDa), common phenomena among the membrane proteins due to their hydrophobic nature with more condensed shapes, leading to a faster migration on the SDS-PAGE (Supplementary data, Figure S1) [35,36]. It was reported that they can run ~70–85% of their estimated molecular mass [37].

**Figure 2:**
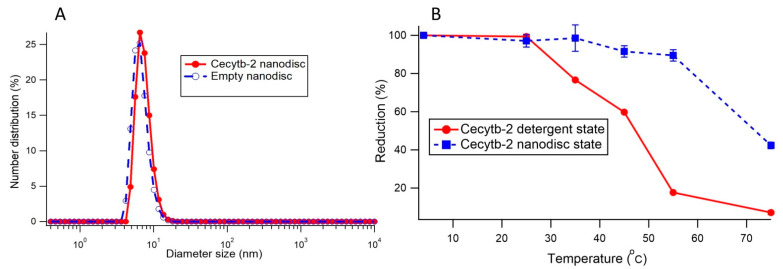
(**A**) Number based size distribution of the SEC-purified Cecytb-2-nanodisc measured by dynamic light scattering (DLS). The red line shows the number distribution of Cecytb-2-nanodiscs and the blue dashed line indicates the number distribution of the MSP1D1ΔH5 empty nanodisc. (**B**) Comparison of the thermal stability of Cecytb-2 in different states. The red line shows the heme reduction level (%) with sodium dithionite for Cecytb-2 in the DDM micelle state and the blue dashed one indicates the heme reduction level (%) with sodium dithionite for Cecytb-2 in a nanodisc state after incubation at different temperatures. (The data were expressed as mean± SD for triplicated experiments. The error bars for the detergent micelle state were smaller than the symbols used).

Due to the strong hydrophobic nature of Cecytb-2 protein, like other CYB561, it was necessary to use a mild detergent (like DDM) to maintain the protein in a solubilized state. However, presence of detergents in the solution would hamper their functions by altering their 3D structure or by dropping their stability. Accordingly, reconstitution of Cecytb-2 into phospholipid bilayer environments (i.e., nanodisc) might be the best solution to elucidate its physiological role as a ferric reductase. Thus, we decided to reconstitute the purified Cecytb-2 protein in a nanodisc, which consists of DMPC as phospholipid bilayer and MSP1D1ΔH5 as a membrane scaffold protein. First, we tried to find the best ratio between DMPC and MSP1D1ΔH5 with a fixed concentration of Cecytb-2. Then, we changed the concentration of Cecytb-2 to find the optimum mixing ratio. We found the best ratio as 1:80:0.5 for MSP, DMPC, and Cecytb-2, which gave the nanodisc complex with minimal aggregations and the highest reconstitution percent. The chromatographic analyses of the resultant Cecytb-2-nanodisc complex on SEC showed a single and sharp symmetrical peak with a retention volume of 13.1 (±0.06) mL and a distribution coefficient (*K_av_*) of 0.36. *K_av_* was calculated by the equation, *K_av_* = (*V*_e_ − *V*_o_)/(*V*_c_ − *V*_o_), where *V*_e_ = elution volume, *V*_o_ = column void volume, *V*_c_ = geometric column volume (Figure 1C). UV-visible spectra of the collected peak fractions of the Cecytb-2 nanodisc (Figure 1D) were used to estimate the physical composition and stoichiometry of incorporated Cecytb-2 into a MSP1D1ΔH5 nanodisc. Analyses on the absorbance at 280 nm and the Soret band peak indicated that the purified Cecytb-2-nanodisc has a stoichiometry of 2.45 (±0.38) MSP1D1ΔH5 to one Cecytb-2 molecule. Furthermore, the heme-reducibility of the Cecytb-2 nanodisc by ascorbic acid (92.68 (±4.17)%) was higher than that in the DDM micelle state (84.5 (±7.8)%), indicating that the Cecytb-2 protein molecule was inserted in native-like environments. The self-assembly process of Cecytb-2 into a phospholipid bilayer nanodisc was highly reproducible, with a mean reconstitution yield of 63.3 (±14.9) % based on the total Cecytb-2 concentration and a reconstitution percent of 83 (±13) % based on the theoretical molar ratio of Cecytb-2 and MSP1D1ΔH5 as 2:1; i.e., one molecule of Cecytb-2 might be incorporated into one nanodisc complex. This assumption was made based our previous results [23] and similar experiments conducted by other groups. Coomassie Brilliant-Blue-stained SDS-PAGE of the purified fraction of the Cecytb-2-nanodisc complex (Figure 1E) revealed that two protein bands corresponding to Cecytb-2 and MSP1D1ΔH5 exist, where the intensity ratio of the two bands, estimated by Image J analysis, was found as 1:1.9, being consistent with those obtained from the analyses on the UV-visible spectra. DLS measurements provided further information about the nanodisc size. Figure 2A indicated that the average size of purified Cecytb-2 nanodisc after SEC was 7.42 (±0.35) nm, which was larger than the empty one (6.72 (±0.20) nm). Moreover, number-based distribution analysis on the DLS data showed a single population with a polydispersity index (PDI) of 0.49, calculated from cumulant analysis to give the overall distribution of the sample, suggesting a nearly monodispersed nanodisc [38]. 

Then, we conducted analyses on the protein thermal stability by incubating Cecytb-2 protein either in a DDM-detergent micelle state or in a nanodisc state at different temperatures. The protein stability was evaluated by the reducibility of heme moiety with sodium dithionite. We observed that there was a notable decrease in the temperature-dependent protein stability for Cecytb-2 in the detergent micelle state, as indicated by the clear decrease in the reduction level at higher temperatures (Figure 2B, red line). By contrast, the nanodisc environments gave much higher protein stability than the detergent micelle state did, as evidenced by the observation that an increase in the incubating temperature up to 55 °C did not cause any significant change in the reduction level (Figure 2B, blue dashed line). 

### 3.2. Ferric Reductase Activity of Cecytb-2 in DDM Detergent State

**Figure 3:**
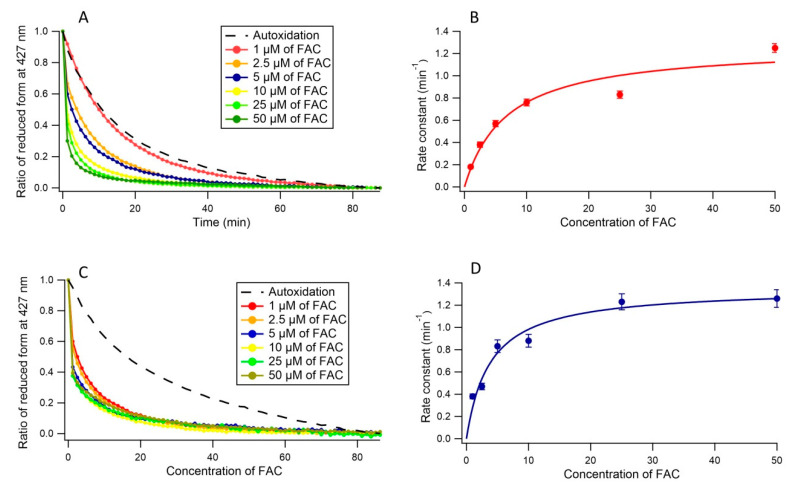
The ferric reductase activity of Cecytb-2 in the DDM detergent micelle state and in a nanodisc state. (**A**) Oxidation of the ferrous heme of reduced Cecytb-2 in DDM detergent micelle state determined by the changes in absorbance at 427 nm after anaerobic addition of FAC and their fittings using a double exponential function; y = y_0_ + A_1_ exp(−*k*_1_t) + A_2_ exp(−*k*_2_t) (for +FAC) and a single exponential function; y = y_0_ + A_1_ exp(−*k*t) (for autoxidation). (**B**) Plot of the rate constants (*k*_2_) from the data shown in panel A against FAC concentrations and its fitting with the Michaelis–Menten Equation. (**C**) Oxidation of the ferrous heme of reduced Cecytb-2 in the nanodisc state determined by the changes in absorbance at 427 nm after anaerobic addition of FAC and their fittings in the same manner as for the detergent micelles state. (**D**) Plot of the rate constants (*k*_2_) from the data shown in panel C against FAC concentrations and its fitting with the Michaelis–Menten Equation in the same manner as for the detergent micelle state.

In order to clarify the ferric reductase activity of Cecytb-2 protein, we measured the autoxidation of the ferrous heme of Cecytb-2 under anaerobic conditions. The measurements showed a very slow decay of the reduced heme (*k* = 0.055 (±0.001) min^−1^) (Supplementary data, Figure S2). This analysis confirmed that the reduced form of Cecytb-2 in the DDM detergent micelle state is stable enough to conduct the measurements of the ferric reductase activity. A significant increase in the oxidation rate of the reduced heme of Cecytb-2 upon anaerobic addition of FAC (Figure 3A) was observed. The acceleration in the oxidation rate was dependent on the concentration of FAC, suggesting that FAC might be working as a substrate for Cecytb-2. Therefore, the acceleration process was examined by fitting with a double exponential function, where *k*_1_ was fixed as the same value with the *k* of the heme autoxidation process, whereas the other rate constant (*k*_2_) was considered as reflecting the ferric reductase activity for FAC. Being consistent with this assumption, the Michaelis–Menten analysis on *k*_2_ and the FAC concentrations showed a hyperbolic saturation with *V*_max_ = 1.27 (±0.139) min^−1^ and *K*_m_ = 6.75(±2.3) µM; *V*_max_/ *K*_m_ = 0.188 min^−1^/µM, as shown in Figure 3B.

### 3.3. Ferric Reductase Activity of Cecytb-2 in the Nanodisc State

The heme-autoxidation of Cecytb-2 nanodisc was determined similarly as in DDM-detergent micelle state. Analysis showed that the oxidation of the ferrous heme in the nanodisc state was slightly slower than in the DDM micelle state, with a rate constant of *k* = 0.035 (±0.008) min^−1^ (Supplementary data Figure S2). Then, we measured the oxidation rate of the reduced heme upon anaerobic addition of FAC in different concentrations as shown in Figure 3C. The kinetic analysis for Cecytb-2 nanodisc (*V*_max_ = 1.35 (±0.087) min^−1^ and *K*_m_ = 3.77 (±0.88) µM; *V*_max_/ *K*_m_ = 0.358 min^−1^/µM) indicated that the Cecytb-2 in the nanodisc state has a higher affinity toward FAC as a substrate than in the DDM micelle state (Figure 3D). We speculated that the ferric substrate FAC could bind to the active site of Cecytb-2 in the nanodisc state with a higher affinity than those in the DDM micelle state. This result suggests that the nanodisc environments can provide much better media for Cecytb-2 protein than in the DDM detergent micelle state by modulating the active site structure slightly. 

### 3.4. Ferric Reductase Activity of Cecytb-2 in Nanodisc and DDM Detergent State Measured by Nitroso-PSAP Assay

**Figure 4:**
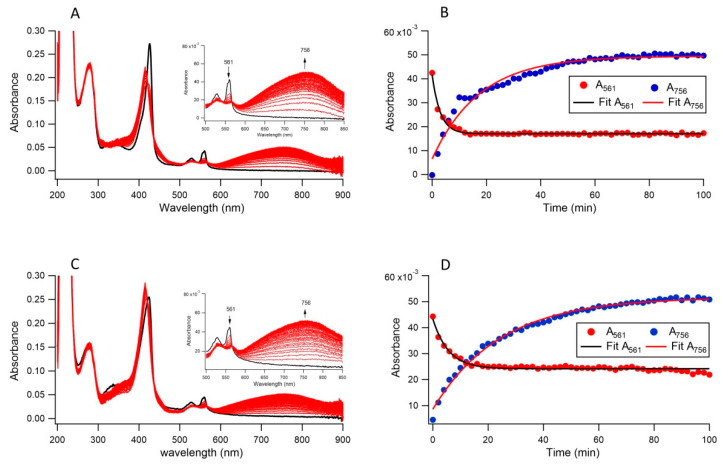
The ferric reductase activity of Cecytb-2 in DDM detergent micelle state and in nanodisc state by using nitroso-PSAP. (**A**) Spectral changes of the reduced Cecytb-2 in nanodisc after simultaneous additions of FAC and nitroso-PSAP anaerobically; black line, fully-reduced Cecytb-2 nanodisc; red lines, following repeated scans upon anaerobic additions of FAC and nitroso-PSAP. The inset shows the enlarged view for the changes in absorbance at 561 nm and 756 nm. (**B**) Plots of the changes in absorbance at 561 nm for the reduced form of Cecytb-2 in nanodisc and at 756 nm for the formation of Fe^2+^-nitroso-PSAP complex against time. Their fittings were conducted with a single exponential function: y = y_0_ + A_1_ × exp(−*k*_1_t). (**C**) Spectral changes of the reduced Cecytb-2 in DDM micelle state; black line, fully reduced Cecytb-2 in the DDM micelle state; red lines, following repeated scans upon anaerobic additions of FAC and nitroso-PSAP. The inset shows the enlarged view for the changes in absorbance at 561 nm and 756 nm. (**D**) Plots of the changes in absorbance at 561 nm for the reduced form of Cecytb-2 in the DDM micelle state and at 756 nm for the formation of Fe^2+^-nitroso-PSAP complex against time. Their fittings were conducted with a single exponential function: y = y_0_ + A_1_ × exp(−*k*_1_t).

The ferric reductase activities of Cecytb-2 in nanodisc and in DDM micelle states were further analyzed using a nitroso-PSAP reagent, which is very sensitive and selective for ferrous iron to form a Fe^2+^-nitroso-PSAP complex giving absorption peak at 756 nm with a molar absorptivity of 45 mM^−1^cm^−1^. Anaerobic addition of nitroso-PSAP and FAC to the reduced form of Cecytb-2 in nanodisc lead to a quick decrease in α band (561 nm) intensity (*k* = 0.33 (±0.014) min^−1^) accompanied by a slightly slower increase in the absorbance at 756 nm (*k* = 0.06 (±0.0035) min^−1^). In the DDM micelle state, a fast decrease in α band intensity (*k* = 0.177 (±0.01) min^−1^) and a slower increase in the absorbance at 756 nm (*k* = 0.04 (±0.001) min^−1^) were observed (Figure 4). These data suggest that the Cecytb-2 in nanodisc state has a higher ability to reduce ferric substrate FAC than in the DDM micelle state at the substrate binding site to form Fe^2+^ ion, which would be released from the active site and react with nitroso-PSAP to form a chromogenic chelate compound (Fe^2+^-nitroso-PSAP complex). The stoichiometric analysis on the ratio of reduced heme *b* vs. Fe^2+^-nitroso-PSAP complex showed 2:1.13 for DDM micelle state and 2:1.7 for the nanodisc state, indicating that a major part of the electrons residing in two hemes *b* centers were transferred to the Fe^2+^-nitroso-PSAP complex via a possible transient intermediate(s).

## 4. Discussion

It was reported that the ascorbic acid (AsA) can act as an electron donor for the members of the CYB561 family [39]. Since CYB561 are trans-membrane proteins and contain two heme *b* prosthetic groups on each side of the membranes [40], the electrons are conveyed between the hemes via a transmembrane electron transfer. Indeed, it was reported that Dcytb has an ability to reduce the extracellular ferric ions through accepting electron from the cytosolic AsA [10], as found originally for neuroendocrine cytochrome *b*_561_ [30,40], which transfers the electrons from cytosolic AsA to re-generate intravesicular AsA. Our previous and present studies were able to show that the Cecytb-2 protein, a Dcytb homolog in *C. elegans*, has an ability to accept electrons from AsA and transferred them to ferric substrates (Miura et al., unpublished results; Fujimura et al., unpublished results). This activity might be accomplished by the presence of two *b*-type heme with different midpoint potentials for each other (and accordingly different functions) (Fukuzawa et al., unpublished results). It is known that detergent solubilization of the integral membrane proteins may hamper their functions by irreversible conformational changes, by forming aggregations, and by lowering protein stability [41]. To avoid these problems, we have succeeded in the reconstitution of the purified Cecytb-2 protein into a MSP1D1ΔH5 nanodisc using a self-assembly method. Our present results showed the feasibility of nanodiscs as a solubilization method to provide native-like environments for very hydrophobic integral membrane proteins, like Cecytb-2. The optimum mixing ratio among Cecytb-2, DMPC, and MSP1D1ΔH5 could produce a homogenous and size-controlled nanodisc, as indicated by SEC measurements as a single peak. Our present results are in full agreement with Bayburt et al. [32], who succeeded in incorporating bacteriorhodopsin (bR) into a nanodisc with a highly reproducible self-assembly process. Our DLS measurements on the purified Cecytb-2-nanodisc showed a homogenous and size-controlled nanodisc. Moreover, the calculated hydrodynamic diameter of the Cecytb-2 nanodisc based on the DLS measurements was slightly larger than the empty nanodisc. This increase in size can be ascribed to the presence of hydrophilic parts of Cecytb-2 protruding from the disc surface that might increase the hydrodynamic volume of the Cecytb-2-nanodisc structure. Our present results agreed well with previous studies using DLS to determine the MSP1D1ΔH5 and MSP1D1 nanodisc [42,43]. Importantly, our present findings and others [44,45] suggested that the protein stability in the nanodisc state was much higher than those in the detergent micelle state, even at higher temperatures. Ravula et al. [20] reconstituted cytochromes P450 and *b*_5_ into a size-controlled nanodisc (~8 nm in diameter) based on the DLS measurements. They indicated that the formed nanodisc was stable for several days at room temperature in full agreement with our results. Further, our results agreed well with Barnaba et al. [19], who indicated that the thermal stability of CYP2B4 was improved after incorporation into a 4F-ER nanodisc.

In the present study, we confirmed that ferric reductase activity of Cecytb-2 by analyzing the oxidation of ferrous heme upon addition of ferric substrate under anaerobic conditions, where clear and saturable dependencies on the substrate concentrations following the Michaelis–Menten equation were observed. Most importantly, we observed a significant enhancement of the ferric reductase activity upon the reconstitution of Cecytb-2 in nanodisc; *V*_max_/*K*_m_ = 0.188 min^−1^/µM for DDM detergent micelle state, and *V*_max_/*K*_m_ = 0.358 min^−1^/µM for the nanodisc state.

Furthermore, we employed another method for analyzing the ferric reductase activity of Cecytb-2. It utilizes anaerobic and simultaneous addition of the nitroso-PSAP reagent and ferric substrate FAC to the reduced form of Cecytb-2 (either in nanodisc or in DDM micelle state). We analyzed the absorbance decay at 561 nm of the reduced heme *b* and the growth of a broad band at 756 nm due to the formation of Fe^2+^-nitroso-PSAP complex simultaneously. It is noteworthy that the rate of electron transfer from the reduced heme to FAC substrate in nanodis state (*k* = 0.33 min^−1^) was faster than in DDM micelle state (*k* = 0.177 min^−1^). Furthermore, the formation of Fe^2+^-nitroso-PSAP complex in the nanodisc state (*k* = 0.06 min^−1^) was slightly faster than in DDM micelle state (0.04 min^−1^). Based on these results, we concluded that the ferric reductase activity of Cecytb-2 was enhanced in the nanodisc state and that nanodisc can provide more suitable environments than detergent micelle. It was notable that the decrease in absorbance at 561 nm was faster than the formation of the 756 nm band in both the DDM micelle and the nanodisc states, suggesting that a possible intermediate product, Fe^2+^-ammonium citrate, resulting from this enzymatic process, is relatively stable within the substrate-binding site. Release of the intermediate product from the active site will depend on the protein conformational dynamics and the conformation of the intermediate at the substrate-binding site. In the present case, the release of Fe^2+^ from the ammonium citrate moiety and the binding with nitroso-PSAP could be relatively slow, resulting in the slow process for the formation of the final Fe^2+^-nitroso-PSAP complex. 

We showed previously that Cecytb-2 protein coded by the *F39G3.5* gene was found to be specifically expressed in the digestive organs of *C. elegans* (Miura et al., unpublished results). Furthermore, when the purified Cecytb-2 protein was reconstituted into AsA-capsuled proteoliposme, it showed a plausible ferric reductase activity upon additions of ferric ion (FeCl_3_) and PDTS (ferrozine) on the extravesicular side (Miura et al., unpublished results). These previous results suggested strongly that Cecytb-2 protein might function as a ferric reductase in the digestive organ of *C. elegans* as a homolog of human Dcytb. The present study confirmed directly the authenticity of the ferric reductase activity of purified Cecytb-2 in the detergent micelle state and in the nanodisc state. The current study might have great significance in understanding of the function of Cecytb-2 and their roles in iron metabolism in *C. elegans*.

Cell-surface assay by adding ferrozine reagent in the medium of the cell culture is a common method to detect the ferric reductase activity of the cells, in which the target proteins were heterologously expressed in host cells (like yeast cells or *Xenopus* oocytes) [10,46]. Ferrozine would chelate ferrous ion produced by cell-surface ferric reductases and a resultant Fe^2+^-ferrozine complex would give a distinct peak at 562 nm [7,47]. In our present study, we did not use ferrozine reagent since our present analyzing method depends deeply on measuring the decay of the ferrous hemes of Cecytb-2 upon the anaerobic addition of ferric substrate FAC and, accordingly, spectroscopic measurements of the α-band peak at 561 nm of Cecytb-2 might be interfered with the absorption peak of the Fe^2+^-ferrozine complex.

Previous studies by using this kinds of cell-surface assay reported that Dcyt*b* [47], mouse cytochrome *b*_561_, fly stromal cell-derived receptor 2 (SDR2) and mouse SDR2 (all these are members of CYB561 protein family) have a ferric reductase activity more or less. Since these belong to different subgroups of the CYB561 protein family, it may be inferred that most of the members of CYB561 protein family have a ferric reductase activity [10,46] and have some roles for the iron transport. The expression sites and expression levels of the CYB561 family members in each organism may provide more information on whether they are actually involved in the iron uptake or not. Dcytb was found to be highly expressed in the duodenum, the main site for iron uptake into the body, but not detected in the liver. In contrast, mouse SDR2 was highly expressed in the liver, which is the main iron storage site. These observations may suggest that the mouse SDR2 has some roles in iron metabolism [48,49]. 

Further studies should be performed to clarify the Cecytb-2 structure and its functions in the nanodisc environment as well as in the detergent micelle state. For studying highly hydrophobic membrane proteins, like Cecytb-2, this study could show the feasibility of the nanodisc technology for solubilization in native-like environments and for elucidating their physiological functions. 

## 5. Conclusions

We found that the reconstituted Cecytb-2 in nanodiscs was extremely stable and more reducible with ascorbate than in the detergent-micelle state. We confirmed the ferric reductase activity of Cecytb-2 by analyzing the enhanced oxidation of ferrous heme upon addition of ferric substrate, where clear and saturable dependencies on the substrate concentrations by following the Michaelis–Menten equation were observed. Further, we confirmed that the ferric substrate was converted to a ferrous state by using a nitroso-PSAP reagent. Importantly, we observed that the ferric reductase activity of Cecytb-2 became enhanced in the phospholipid bilayer environments. 

## Figures and Tables

**Table 1:** The purification of Cecytb-2-His_6_ by Ni- Sepharose affinity chromatography.

Sample	Dithionite-Reducible *b*_561_ Content (nmoles)	Total Protein (mg)	Specific Content (nmole/mg)	Yield (%)	Fold
DDM-solubilized fraction	718.2	382.64	1.88	100	1
Ni-NTA Sepharose fraction	162.2	4.148	39.1	22.58	21.07

## Data Availability

Data will be available upon request.

## Supplementary Materials

The following are available online at https://www.mdpi.com/2218-273X/11/1/96/s1; Figure S1 provides a SDS-PAGE of the purified Cecytb-2-nanodisc complex after SEC. Figure S2 provides a comparison of the autoxidation process of the ferrous heme of fully reduced Cecytb-2 protein in DDM-detergent micelle state and in nanodisc state.
