# Supplementary material for: Functional Assembly of Caenorhabditis elegans Cytochrome b-2 (Cecytb-2) into Phospholipid Bilayer Nanodisc with Enhanced Iron Reductase Activity
Source: Biomolecules. 2021 Jan 13;11(1):96. doi: 10.3390/biom11010096 (PMC7828500; doi:10.3390/biom11010096)
Supplement: Supplementary file 1 [file biomolecules-11-00096-s001.pdf]

# Supplemental data for
# Functional assembly of *Caenorhabditis elegans* cytochrome b-2 (Cecytb-2) into phospholipid bilayer nanodisc with enhanced iron reductase activity

**Hamed A. Aboshararaf[1,2,*], Yuki Sakamoto[1], Aliaa M. Radwan[1,2], Keisuke Yuzu[1], Mika Fujimura[1], Thoria Diab[2], Tarek M. Mohamed[2], Eri Chatani[1], Tetsunari Kimura[1], Motonari Tsubaki[1,*]**

[1] Department of Chemistry, Graduate School of Science, Kobe University, Nada-ku, Kobe, Hyogo 657-8501, Japan; ac_for_npi@yahoo.co.jp (Y.S.); alyaa_radwan@science.tanta.edu.eg (A.M.R.); 192s227s@stu.kobe-u.ac.jp (K.Y.); mika-fujimura@unicharm.com (M.F.); chatani@crystal.kobe-u.ac.jp (E.C.); tetsunari.kimura@people.kobe-u.ac.jp (T.K.)

[2] Biochemistry Division, Chemistry Department, Faculty of Science, Tanta University, Tanta 31527, Egypt; thoria.diab@science.tanta.edu.eg (T.D.); tarek.ali@science.tanta.edu.eg (T.M.M.)

* Correspondence: hamed_biochemistry@science.tanta.edu.eg (H.A.A.); mtsubaki@kobe-u.ac.jp (M.T.)

## Expression of Cecytb-2 in *Pichia pastoris* cells

A single colony of *Pichia pastoris* GS115/ pPICZb-Cecytb-2+His6 was inoculated into 25 mL of buffered minimal glycerol complex media (BMGY) and incubated overnight at 30 °C with shaking at 220 rpm. Then, the cells were transferred to 225 mL of main BMGY medium (1% yeast extract, 2% tryptone, 100 mM potassium phosphate buffer, pH 6, 2% glycerol, 1.34% yeast nitrogen base, 0.004 % L-histidine, $4x10^{-5}$ % biotine , 25 µg/ mL Zeocin) and incubated at 30 °C for 48 h with shaking at 220 rpm. Then the cells were harvested by centrifugation at 8000 rpm for 4 min, at room temperature, and suspended in 250 mL of BMMY (the same BMGY but contains 2% methanol without glycerol and zeocin) for induction and incubated at 30 °C for 96 h with shaking at 220 rpm. However, methanol and histidine were added to BMMY medium with a final concentration of 2% and 40 µg/mL, respectively every 24 h during the induction period. At the end of the induction, the cells were harvested by centrifugation (8000 rpm for 4 min at 4 °C). The pellet was suspended in buffer A (50 mM potassium phosphate buffer, pH 7, 2 M sorbitol, 0.1 mM EDTA-Na, pH 8, 0.1 mM dithiothreitol). Then, zymolase (from *Arthrobacter luteus*) (Nacalai Tesque, Inc., Kyoto, Japan) was directly added to the suspension to make a final

concentration of 1 mg per 15 g of the cells and the suspension was shaken at 35 °C, 180 rpm for 16 h.

### Preparation of *Pichia pastoris* microsomal fraction

All following steps were carried out on ice with using ice-cold buffers. The spheroplast pellet after the zymolyase treatment was recovered by centrifugation at 4 °C, 18,000 rpm, 15 min and re-suspended in buffer B (50 mM potassium phosphate buffer, pH 7, 0.65 M sorbitol, 0.1 mM EDTA-Na, 0.1 mM dithiothreitol, 1mM PMSF). Then, the suspension was sonicated with an Astrason S3000 ultrasonic processor (Misonix Inc., Farmingdale, NY) at 0.7 pulse, off time 2.0 sec., power 8.0 with stirring in an ice-cold condition, followed by centrifuged at 4 °C, 18,000 rpm, for 15 min. Thus obtained supernatant was ultra-centrifuged at 4 °C, 30,000 rpm for 1 h to collect microsomal fraction. The microsomal fraction was re-suspended in buffer C (50 mM potassium phosphate buffer, pH 7, 10% glycerol) and was stored at −80 °C until use.

### Microsomal membrane solubilization and purification of Cecytb-2

The frozen microsome was thawed and solubilized in solubilization buffer (50 mM potassium phosphate buffer, pH 7, 10% glycerol, 1.5 % DDM, 1 mM PMSF, 20 mM imidazole, 300 mM NaCl) with stirring for 1h. Then, the solubilized fraction collected by ultra-centrifugation at 30000 rpm for 30 min. at 4 °C and loaded onto a Ni-Sepharose column (GE Healthcare Bio-sciences AB, Uppsala, Sweden) pre-equilibrated with buffer A (50 mM potassium phosphate buffer, pH 7.4, 10% glycerol, 0.1 % DDM, 20 mM imidazole, 300 mM NaCl). Then, the attached His-tagged Cecytb-2 to the Ni-Sepharose resin was washed successively with buffers containing different imidazole concentrations (20, 40, 60 and 80 mM), followed by elution with an elution buffer (50 mM potassium phosphate buffer, pH 7.4, 10% glycerol, 0.1 % DDM, 350 mM imidazole, 300 mM NaCl). Then, imidazole in the eluate was removed by gel filtration using a PD-10 column (GE Healthcare, UK) equilibrated with 50 mM potassium phosphate buffer, pH 7.4, 10% glycerol, 0.1 % DDM.

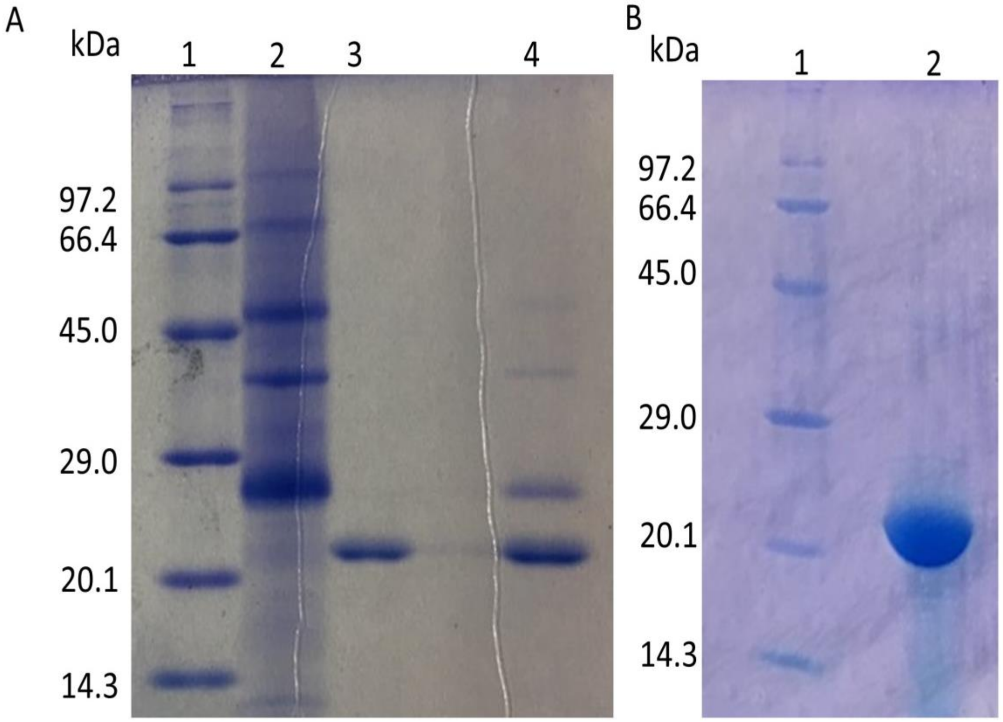


Figure S1. (A) SDS-PAGE of the purified Cecytb-2-nanodisc complex after SEC. Lane (1) protein marker (LMW marker (SP-0110), APRO Life Science Institute, Inc, Osaka, Japan); lane (2) purified Cecytb-2; lane (3) empty MSP1D1ΔH5 nanodisc; lane (4) Cecytb-2 nanodisc (Cecytb-2, 29.2 kDa and MSP1D1ΔH5, 22.1 kDa). (B) SDS-PAGE of the purified MSP1D1 ΔH5 after Ni-Sepharose column purification. Here we used a protein marker different from that used in Fig. 1 (main text) showing a more reasonable migration on SDS-PAGE.

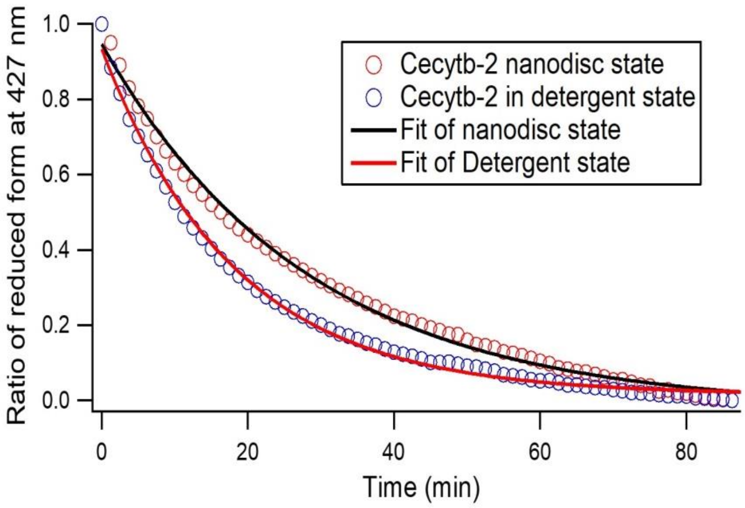


Figure S2. Comparison of the autoxidation process of the ferrous heme of fully reduced Cecytb-2 protein in DDM-detergent micelle state and in nanodisc state. Blue line; Cecytb-2 in nanodisc; Broken red line, Cecytb-2 in DDM-detergent micelle state. Cecytb-2 in each state was reduced by sodium dithionite and loaded onto the HiTrap™ desalting column anaerobically and eluted by deoxygenated elution buffer (50 mM Tris-HCl, 10% glycerol) into deoxygenated cuvette. Then their UV-visible spectra were measured by a repeated scan mode at room temperature. The absorbance change at 427 nm was plotted against time and fitted by a single exponential function; $y = y_0 + A_1 \exp(-k_1 t)$.
